# Experiments Bearing on the Infliction of the Death Penalty by Electricity*Extract from a report of the Commission appointed by the Governor of New York to investigate and report on the most humane and practical method of carrying into effect the sentence of death in capital cases.

**Published:** 1888-02

**Authors:** George E. Fell

**Affiliations:** Professor of Physiology and Microscopy, Medical Department of Niagara University, Buffalo, N. Y.


### *EXPERIMENTS BEARING ON THE INFLICTION OF THE DEATH PENALTY BY ELECTRICITY.**

By GEORGE E. FELL, M D., F. R. M. S.,

Professor of Physiology and Microscopy, Medical Department of Niagara University, Buffalo, N. Y.

In the month of July, 1887, there was conducted a series oi experiments calculated to throw considerable light upon the value of electricity in killing dogs. The authorities of the city of Buffalo, N. Y., had determined to rid the city of the numerous curs roaming the streets. To reduce their sufferings to a minimum, the agent of the Society for the Prevention of Cruelty to Animals recommended that electricity be applied as the death-dealing agent. The experiments were conducted at the improvised dog-pound, prepared at old Police Headquarters. The canines were quartered in one room; adjoining this was an entry which communicated with a third room, in which the electrical apparatus was located.

### THE ELECTRICAL APPARATUS.

This consisted of a common pine box, lined with zinc, and connected with one pole of the electric-light current for that portion of the city. When in use, the box was partially filled with water. Connected with the electric-light wire, representing the other pole, was an ordinary dog-muzzle, supplied with an iron or copper bit, which was inserted into the mouth of the canine.

The animal being placed in the box, the switch making the circuit was turned, causing the apparent instantaneous death of the animal. Only in exceptional cases were any movements noted after the current was made.

The results obtained by experiments conducted in this manner leaves the subject just where public opinion would place it, viz., that electricity will kill quickly. However, to ascertain how quickly and thoroughly, requires further demonstration.

* Extract from a report of the Commission appointed by the Governor of New York to investigate and report on the most humane and practical method of carrying into effect the sentence of death in capital cases.

The heart may rightfully be considered the center of function, and, in the execution of criminals by the legalized hanging process, is always examined to ascertain when death ensues. In favorable cases, it is known that the heart may beat from six to ten minutes, and in some cases it has been known to beat from fifteen to thirty minutes before death.

For the purpose of ascertaining the effect of the electric-light current on the action of the heart, the operation of opening the thorax of an animal under forced respiration was made. With this operation satisfactorily performed, the heart and lungs may be observed in action, viz., the heart beating and the lungs contracting and expanding as in life.

While the operation is not new to physiologists, still the effect upon the movements of the heart, by the passage of an electrical current which might be applied in the execution of criminals, I do not believe, has been frequently noted, or the operation often performed. That the ordinary electric-light current used in these experiments is sufficient to cause instantaneous death of a human being, is inferred from the many accidental deaths produced by such means. To witness the effect produced upon the heart in action, is a demonstration which cannot be questioned, and offers a positive answer to what may have been inferred, and has already amounted to, a foregone conclusion.

To those favoring electricity as the proper agent in the execution of criminals, a demonstration of this character serves to make them more positive, and less liable to be influenced by those whose investigations have been only superficial.

Those opposed to it from the standpoint of uncertainty of action, it leaves without a foundation upon which to base their opinion. Prior to these experiments, I held the view that electricity might prove the best agent for executing criminals; after they were made, I enthusiastically supported it as the only agent which this age had any right to use for this purpose.

But to refer to the experiments: A fair-sized dog was placed under the influence of chloroform; an incision was made in the trachea, in which a tube, connecting with the foot-bellows, and supplied with suitable valve for respiratory purposes, was attached. Repirations were then kept up by these artificial means. The chest walls (thorax) were then removed, so that the heart and lungs were exposed to view; the dog was then placed in the zinc-lined box, the muzzle put on, and the forced respiration kept up until just before the current was made. The heart was beating as in life, but, the instant the circuit was made, it ceased its action, and became a mere mass of quivering flesh; not the least resemblance to a rythmical movement was observed after the current was made. Many citizens present at the operation can testify to the above; in fact, the demonstration as to suddenness of stoppage of the heart exceeded all anticipation. The interference with all function was electrically instantaneous; death ensued from electrical shock; the ordinary conditions of dying were absent. Nothing could be more sudden.

This first experiment, although eminently satisfactory in its results, was made under conditions the most unsatisfactory. The room was full of men hurrying to and fro, the dogs were being led to their fate, and no suitable place was provided to operate. This, and observations connected with another series of investigations, led to another demonstration, which was made under more favorable conditions, at the electric-light works, on Wilkeson street. This operation was conducted with greater care; instead of the muzzle with the bit attached, to make one pole of the circuit, a piece of wire line was placed in the mouth of the dog, and wound around the nose. The zinc box was used as before; chloroform narcosis was produced as before, and the thoracic walls removed. The heart was beating rythmically; on making the circuit, it instantly ceased to beat. The current was quickly turned of, and forced respirations kept up, with the view of bringing the heart again into action; this was entirely unsuccessful. This result demonstrates that if the current is sufficiently powerful, attempts at resuscitation in the case of a criminal executed by electricity would fail.

In this second experiment, it was also noticed that an attempt to respire was made by the animal after the current was made.

This, undoubtedly, indicated that the respiratory center in the brain (medulla) had not completely lost its susceptibility to impressions, and that, through the want of oxygen in the blood and center noted, the effort to breathe was formulated. This has an important bearing upon the apparatus to be used in executions, inasmuch that it indicates that the poles should be arranged to pass the current through the center of function in the brain. Upon physiological grounds, also, this is indicated.

Even without this refinement of precision in the apparatus, as has been shown in this experiment, where the current was not passed directly through the functional brain centers, the sudden stoppage of the heart would indicate that electricity offers the most rapid agent in producing death, that we have at our command. The mere estimates of the difference in the speed of the electric as compared with the nervous current, would further indicate that our senses could not interpret or apprehend the passage, or that death, produced by such means, would be absolutely painless to the culprit.

From observations which are generally accepted, it may be stated that nervous force travels at a rate of from one to two hundred feet per second, while the electric current passes at a rate not less than two hundred thousand feet per second, or about one thousand times as rapid.

From these observations, the following deductions may be drawn :

First—That death, produced by a sufficiently powerful electric current, is the most rapid and humane of that produced by any agent at our command.

Second—That resuscitation after the passage of such a current through the body and functional centers of the brain, is impossible.

Third—That the apparatus to be used should be arranged to permit the current to pass through the centers of function and intelligence in the brain.

